# T-Cell Receptor Profiling and Prognosis After Stereotactic Body Radiation Therapy For Stage I Non-Small-Cell Lung Cancer

**DOI:** 10.3389/fimmu.2021.719285

**Published:** 2021-10-18

**Authors:** Lirong Wu, Jun Zhu, Nils-Petter Rudqvist, James Welsh, Percy Lee, Zhongxing Liao, Ting Xu, Ming Jiang, Xiangzhi Zhu, Xuan Pan, Pansong Li, Zhipeng Zhou, Xia He, Rong Yin, Jifeng Feng

**Affiliations:** ^1^ Department of Radiation Oncology, Jiangsu Cancer Hospital & Jiangsu Institute of Cancer Research & The Affiliated Cancer Hospital of Nanjing Medical University, Nanjing, China; ^2^ Department of Radiation Oncology, University of Texas MD Anderson Cancer Center, Houston, TX, United States; ^3^ Department of Thoracic/Head & Neck Medical Oncology, University of Texas MD Anderson Cancer Center, Houston, TX, United States; ^4^ Department of Immunology, University of Texas MD Anderson Cancer Center, Houston, TX, United States; ^5^ Department of Medical Oncology, Jiangsu Cancer Hospital & Jiangsu Institute of Cancer Research & The Affiliated Cancer Hospital of Nanjing Medical University, Nanjing, China; ^6^ Department of Scientific Research, Geneplus-Beijing Institute, Beijing, China; ^7^ Department of Thoracic Surgery, Jiangsu Cancer Hospital & Jiangsu Institute of Cancer Research & The Affiliated Cancer Hospital of Nanjing Medical University, Nanjing, China; ^8^ Jiangsu Biobank of Clinical Resources, Nanjing, China

**Keywords:** T-cell receptor sequencing, stereotactic body radiation therapy, non-small-cell lung cancer, treatment failure, disease progression

## Abstract

Radiotherapy is known to influence immune function, including T cell receptor (TCR) repertoire. We evaluated the TCR repertoire before and after stereotactic body radiotherapy (SBRT) for stage I non-small-cell lung cancer (NSCLC) and explored correlations between TCR indexes and distant failure after SBRT. TCR repertoires were analyzed in peripheral blood mononuclear cells (PBMCs) collected before and after SBRT from 19 patients. TCR combinational diversity in V and J genes was assessed with multiplex PCR of genomic DNA from PBMCs and tested for associations with clinical response. All patients received definitive SBRT to a biologically effective dose of >=100 Gy. The number of unique TCR clones was decreased after SBRT versus before, but clonality and the Shannon Entropy did not change. Four patients (21%) developed distant metastases after SBRT (median 7 months); those patients had lower Shannon Entropy in post-SBRT samples than patients without metastasis. Patients with a low change in Shannon Entropy from before to after SBRT [(post-SBRT Shannon Entropy minus baseline Shannon)/(baseline Shannon) * 100] had poorer metastasis-free survival than those with high change in Shannon Entropy (P<0.001). Frequencies in V/J gene fragment expression in the TCR β chain were also different for patients with or without metastases (two V fragments in baseline samples and 2 J and 9 V fragments in post-treatment samples). This comprehensive analysis of immune status before and after SBRT showed that quantitative assessments of TCRs can help evaluate prognosis in early-stage NSCLC.

## Introduction

Stereotactic body radiotherapy (SBRT), in which ablative doses of radiation are given in a few large fractions to highly conformal targets, is an established treatment option for the management of stage I medically inoperable non-small cell lung cancer (NSCLC) (1). The excellent local control rates after SBRT for early-stage disease (>90%) are comparable to those after surgery, and the low incidence and severity of short-term or long-term overall toxicity (2) make SBRT a suitable treatment option, especially for patients with poor pulmonary function (3). Several studies have also shown that some patients with oligometastatic NSCLC, that is, those with a few isolated metastases (4), can experience long-term survival when SBRT is used as consolidative therapy (5).

SBRT may trigger both localized and systemic immune responses against tumors. Locally, by directly damaging cancer cells, it leads to antigen exposure, which leads in turn to activation of both local and systemic immune systems (6). SBRT can also induce DNA damage (7), and the resulting mutations in cells with DNA mismatch repair deficiency can increase the burden of neoantigens, which in turn trigger an immune response (8). By sensitizing tumor cells to immunotherapy, radiation causes cells to release further tumor antigens that stimulate T cells and attack other tumor cells in the body, including those at distant, unirradiated sites, thereby turning tumors into “vaccines” (9) that can cause abscopal effects (10, 11). Immunologic effects may be useful as biomarkers that could be used to distinguish patients for whom tumors could be controlled versus those for whom disease progresses after treatment (12).

SBRT is also thought to trigger systemic immune responses *via* radiotherapy-induced microenvironmental changes to both tumor cells and the surrounding stromal cells (13). For immunologically sensitive tumors, SBRT-induced remissions depend on the development of antitumor immunity that is reflected in the tumor cell microenvironment (14). Thus systematic evaluations of the tumor immune microenvironment may help to optimize the ability of radiation therapy to evoke a robust immune response. Because T cells are essential factors in the anticancer immune response, contributing to the cancer cell killing activities of immunotherapy as well as that of traditional treatments such as chemotherapy and radiotherapy (15, 16), an in-depth analysis of T cells may provide essential insights into understanding a particular individual tumor and immune status (17).

T cells recognize antigenic peptides *via* specific receptors expressed on the surface of the T cells. Each T-cell receptor (TCR) is unique and varies within both individuals and populations, allowing effective immune responses to a wide range of foreign antigens (18). The diversity of the TCR repertoire reflects the diversity of cellular immunity, and several studies have shown that diversity in CDR3, a hypervariable or complementarity-determining region (CDR) in the variable domains of the TCR α-chain and β-chain that recognizes processed antigens, is essential in cancer diagnosis, therapy, and prognosis (19–22). TCR sequencing has been used to examine intratumoral T-cell responses in solid cancers, including NSCLC (19, 23). TCR repertoire analysis has also been used as a biomarker in the context of immune checkpoint blockade (24). Liu Y. et al. revealed that increased diversity and high overlap rate between the pre-and post-treatment TCR repertoires were associated with better outcomes in advanced lung cancer (23). Zhang J. et al. reported that higher TCR clonality was associated with reduced percent residual tumor at the time of surgery and exchange of T-cell clones between tumor and blood was important to pathologic response in neoadjuvant immunotherapy (25). However, the role of the TCR repertoire as a biomarker for outcomes after SBRT for stage I NSCLC is unknown at this time. Here, we systematically analyzed CDR3 from the TCRβ chain in peripheral blood mononuclear cell (PBMC) samples from patients with stage I NSCLC obtained before and after SBRT and explored potential correlations between TCR indexes and clinical outcomes after SBRT.

## Materials and Methods

### Patients

Peripheral blood samples were collected and PBMCs isolated from 19 patients with stage I NSCLC from April 2018 through January 2019 at the Jiangsu Cancer Hospital & Jiangsu Institute of Cancer Research & The Affiliated Cancer Hospital of Nanjing Medical University (Nanjing, China). The collection and use of patient samples were approved by the institutional review board, and informed consent was obtained from all patients.

Of the 19 patients, 14 had a newly diagnosed stage I primary lung tumor, and the other 5 had oligometastatic NSCLC after surgical removal of the primary lung tumor. Disease was considered medically inoperable in all patients, either because of other comorbid conditions (n = 12) or because the patients refused surgery (n = 7), and all 19 patients underwent SBRT. Baseline clinical characteristics were collected within the 30 days before treatment initiation. Enhanced computed tomography (CT) and positron emission tomography (PET)/CT scanning confirmed that all patients had the node-negative disease. No patients had a history of other malignancies, and none had had chemotherapy or radiotherapy. Patients were staged according to the 8th edition of the AJCC/UICC staging system and then grouped into with or without metastases after SBRT.

Blood samples were collected at admission before SBRT was begun and again within 24 hours after SBRT was completed from all 19 patients. Radiation treatment planning was based on 4D-CT scans with contrast and 3-mm slices acquired while the patient was in the treatment position. SBRT was delivered in all cases in 3-5 fractions, with a biologically effective dose (BED10) of at least 100 Gy, as either intensity-modulated radiation therapy or volumetric modulated arc therapy, both with image guidance. Clinical information was extracted from the hospital’s case management system, and the clinical response to treatment was evaluated by physicians according to the Response Evaluation Criteria in Solid Tumors (RECIST 1.1).

### Blood Sample Collection

Six-milliliter samples of peripheral venous blood were obtained from all patients in ethylenediaminetetraacetic acid (EDTA)-coated tubes at two time points: once before RT (baseline) and one within 24 hours after SBRT. PBMCs were isolated by Ficoll-gradient centrifugation (Ficoll-Paque Plus, GE Healthcare), and samples were stored at -80°C immediately after collection until further analysis.

### Patient Follow-Up

Patients were examined weekly during treatment, every 3 months during the first year after treatment, every 6 months during the second year, and then annually thereafter. Clinical information on sex, age, smoking history, clinical disease stage, tumor histology, tumor volume, RT dose and BED, and Karnofsky Performance Status (KPS) score were obtained from hospital records. Performance status was measured prospectively for up to 20 months after treatment. The progression-free survival (PFS) interval was the period between treatment initiation to the date of disease progression or death, whichever occurred first.

### DNA Extraction and TCR Sequencing

A total of 38 blood samples were collected from the 19 patients (19 before SBRT and 19 after SBRT) and analyzed for TCR-β sequencing as follows. Genomic DNA was extracted from frozen PBMC specimens by using a High Pure PCR Template Preparation Kit (Roche). Multiplex PCR amplification of the CDR3 region of the TCR β chain was done in two rounds, PCR1 and PCR2. The first round, PCR1, was done with specific primers against each V and J gene. Primer sequences were filed as part of a Chinese patent (CN105087789A). The second round of PCR2 was done with universal primers. We used paired-end sequencing of samples with a read length of 151 bp with the Illumina HiSeq 3000 platform. Raw sequencing data were processed and analyzed as follows. First, the raw data were filtered by using CutAdapt to remove sequences that did not contain the primers for multi-PCR (26). Second, the remaining high-quality paired reads were merged to obtain contigs by using the PEAR (Paired-End read merger) program. Third, Mixcr was used to align the reference TCR β chain V/(D)/J gene sequences to determine the TCR β chain V/(D)/J gene segments in each contig (27). Finally, the CDR3 species were clustered to eliminate sequencing errors according to base quality and sequence similarity.

The diversity of the TCR repertoire was calculated based on the normalized Shannon entropy index, which was determined by dividing the Shannon entropy index by the natural logarithm of the productive TCR sequence number (28). Clonality was further defined as 1 - (Shannon entropy index)/ln K, where K is the number of unique clonetypes (29).

### Statistical Analysis

Differences among groups were analyzed with Wilcoxon tests. P values were obtained with log-rank tests, and the Kaplan-Meier method was used to estimate progression-free survival. Cut-off values in the Kaplan–Meier survival analysis were identified by using the Youden index of the receiver operating characteristic (ROC) curve. All statistical analyses were calculated by using R (V3.5.3). All P values were unadjusted and A two-tailed P < 0.05 was considered to indicate statistical significance.

## Results

### Subject Characteristics and Outcomes

Patient and disease characteristics are shown in **Table 1**. All 19 enrolled patients had SBRT to a BED of at least 100 as definitive treatment for NSCLC (14 primary tumors and 5 oligometastatic lesions after surgery), and no patients received concurrent therapy. Performance status, tumor volume, and PET standardized uptake values (SUV) were documented at baseline. The median age of the 13 men (68%) and 6 women (32%) was 74.5 years (range 53-84 years). Pathology assessment indicated that tumors were squamous cell carcinoma in 7 patients and adenocarcinoma in 12. The median follow-up time was 23 months (range 5-27 months) for all patients. The median PFS time was 10 months (range 5-17 months). Four patients (21%) developed distant metastases after SBRT, one of whom had been treated for oligometastatic disease and the other 3 for primary tumors; the remaining 15 patients had no significant disease progression. The median time to metastasis was 7 months (range 5-8 months).

**Table 1:** Clinical characteristics of the patients.

Characteristics	Value or No. of Patients (% or Range)
Age, years	
50-64	5 (26)
65-74	5 (26)
75+	9 (47)
Sex	
Male	13 (68)
Female	6 (32)
Tumor Histology	
Squamous cell carcinoma	7 (37)
Adenocarcinoma	12 (63)
Disease Stage (AJCC 8th edn)	
T1b	6 (32)
T1c	9 (47)
T2a	4 (21)
Progression-Free Survival Interval	
<6 months	1 (5)
6-9 months	5 (26)
>9 months	13 (68)
Outcome	
Metastasis	4 (21)
No metastasis	15 (79)
SBRT	
50 Gy in 5 fractions (BED=100)	5 (26)
56 Gy in 7 fractions (BED=101)	14 (74)
Baseline Tumor Volume, median, cm3	18.3 (2.3-76.4)
Baseline PET SUV, median	6.45 (3.9-9.4)
Baseline Karnofsky Performance Status Score, median	90 (80-100)
Tumor Type	
Primary	14 (74)
Recurrent	5 (26)
Smoking Status	
Never	5 (26)
Former	3 (16)
Current	11 (58)
No. Smoking Pack-Years, median	58 (15-114)

AJCC, American Joint Committee on Cancer; SBRT, stereotactic body radiation therapy; BED, biologically effective dose; PET positron emission tomography; SUV, standardized uptake value.

### TCR Repertoire Analysis Before and After SBRT

**Figure 1:**
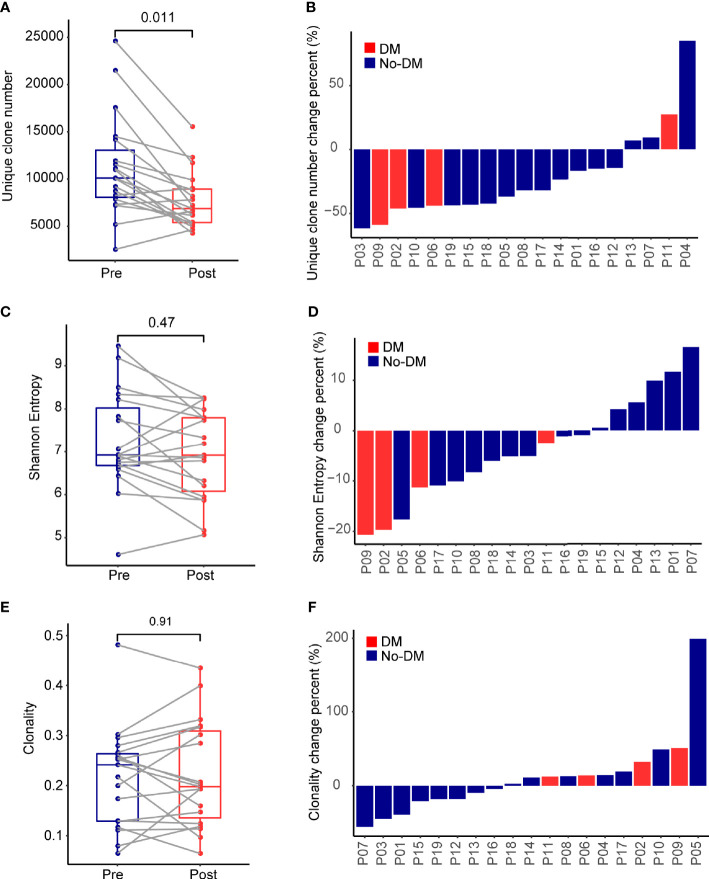
Effects of stereotactic body radiotherapy (SBRT) on the immune repertoire. **(A)** Boxplots show the percentage change in number of unique TCR clones per patient from before (Pre) and after (Post) SBRT. Change is calculated as [(No. of TCR clones after SBRT minus No. of TCR clones before SBRT)/(No. of TCR clones before SBRT)]. **(B)** Waterfall plot showing percent changes in number of unique TCR clones after SBRT versus before SBRT per patient, calculated as described in **(A)**. **(C)** Boxplots show the percentage change in Shannon Entropy per patient [Shannon Entropy after SBRT minus Shannon Entropy before SBRT)/(Shannon Entropy before SBRT)]. **(D)** Waterfall plot showing percent changes in Shannon Entropy per patient, calculated as described in **(C)**. **(E)** Boxplots show the percentage change in clonality per patient from before to after SBRT, calculated as [(Clonality after SBRT minus clonality before SBRT)/(clonality before SBRT)]. **(F)** Waterfall plot showing the percent changes in clonality, calculated as described in **(E)**. P values were calculated with Wilcoxon tests. For each panel, red indicates the 4 patients who developed distant failure (DM) after SBRT, and blue indicates the 15 patients without distant failure (No-DM).

Comprehensive recognition of different antigens by T cells depends on having a diverse TCR repertoire, which can be quantified in terms of numbers of unique clones, the Shannon Entropy, and clonality (30). Genomic DNA extracted from the PBMCs was quantitatively TCR-β-sequenced to assess and compare T-cell clonality from before to after treatment (**Supplementary Figure 1**), and boxplots were used to summarize the change in the number of unique clones. Samples obtained before SBRT had more unique clones (median 10,088, range 2,466-24,618) than did samples obtained after SBRT (median 6,861, range 4,174-15,541) (Wilcoxon paired signed-rank test, P=0.011) (**Figure 1A**). Patients lost a lot of TCR clones (median 48%, range 21%-82%) and developed numerous new TCR clones (median 46%, range 25%-73%) after SBRT (**Supplementary Figure 2**). Analysis of the change in the number of unique clones [calculated as (Post-SBRT TCR clone number - Baseline TCR clone number)/(Baseline TCR clone number)] revealed that only 4 of the 19 patients had more unique clones after SBRT (median 18.2%, range 6.9%-85.0%), whereas the other 15 patients had fewer unique clones after SBRT (median -43.4%, range -62.0% ~ -14.6%) (**Figures 1A, B**). One of the four patients who developed metastases after SBRT had more unique clones after SBRT and the other three patients had fewer unique clones after SBRT. The Shannon Entropy was no difference between the baseline (median 6.92, range 4.61-9.46) and after-SBRT samples (median 6.92, range 5.07-8.26) (P = 0.47) (**Figure 1C**). Specifically, 5 of the 19 patients had a higher Shannon Entropy and 14 had a lower Shannon Entropy after SBRT (**Figure 1D**). Notably, all 4 of the patients who developed metastases after SBRT had lower Shannon Entropyes after SBRT. We further found that clonality was no different among patients who did or did not develop distant metastases after SBRT (P = 0.91) (**Figures 1E, F**). Notably, although the number of unique clones was lower after treatment *vs*. at baseline (**Figure 1A**), the Shannon Entropy was no difference between patients who did or did not develop metastases (**Figure 1C**), implying that some fraction of TCR clones might experience heterogeneous expansion and others might be eliminated under therapeutic pressure.

### Correlation of TCR Repertoire With Metastases After SBRT

**Figure 2:**
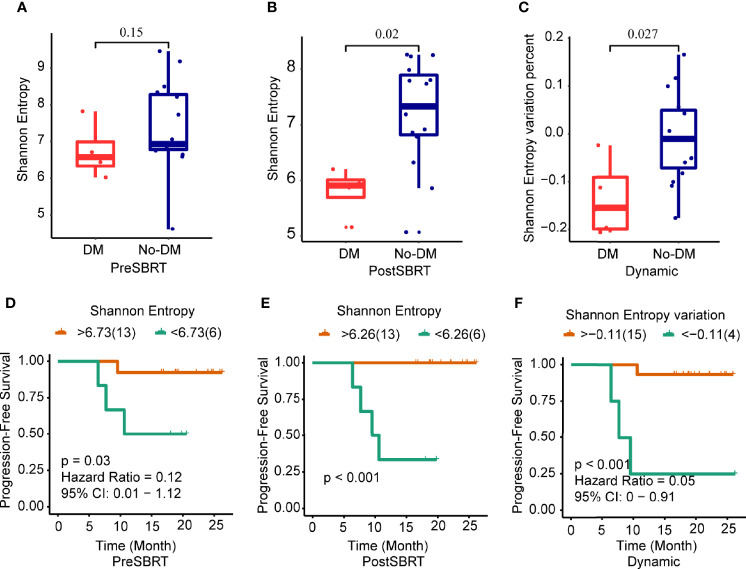
Changes in Shannon Entropy and treatment outcomes after SBRT. **(A−C)** Boxplots showing differences in Shannon Entropyes in baseline (before-SBRT) samples **(A)**; in samples collected after SBRT **(B)**; and in the difference in Shannon Entropy between before and after SBRT [Shannon Entropy after SBRT minus Shannon Entropy before SBRT)/(Shannon Entropy before SBRT)], according to whether patients developed metastases after SBRT (DM) or did not (no-DM). **(D−F)** Kaplan–Meier progression-free survival estimates according to Shannon Entropy cut-off levels for samples obtained before SBRT **(D)**, after SBRT **(E)**, or the percent change in Shannon Entropy from before to after SBRT **(F)**. Patients with high baseline Shannon Entropy (i.e., >6.73) had significantly longer progression-free survival; patients with high post-SBRT Shannon Entropy (>6.26) had longer progression-free survival; and patients with higher percent change in Shannon Entropy (> −0.11) had longer progression-free survival. P values are derived from unadjusted log-rank tests.

Next, to determine whether prognosis after SBRT was associated with changes in the TCR repertoire, we focused on the potential relationship between TCR indexes and distant metastases after SBRT. TCR diversity was estimated with the Shannon Entropy. The Shannon Entropy at baseline was not associated with metastases (**Figure 2A**). However, the Shannon Entropy after SBRT was significantly lower in the patients who developed distant metastases than in those who did not (**Figure 2B**). Moreover, the difference in Shannon Entropy was lower for patients who developed distant metastases than for those who did not (P=0.027) (**Figure 2C**).

Next, we examined the ROC curves to identify the optimal cut-points for the baseline Shannon Entropy, the after-SBRT Shannon Entropy, and the change in Shannon Entropy (post/baseline) (**Supplementary Figures 3A-C**). Measures of TCR diversity and paired samples were combined to classify patients as having either “good” (low-risk) or “poor” (high-risk) immune status. The optimal cut-off point for baseline Shannon Entropy in terms of PFS was 6.73; the good or low-risk group (baseline Shannon Entropy >6.73) had better PFS than the poor or high-risk group (<6.73) (P=0.03) (**Figure 2D**). As for the after-SBRT Shannon Entropy, the cut-off points of 6.26 distinguished patients as having poor or high-risk immune status (<6.26) versus good or low-risk immune status (>6.26), and values correlated with PFS (P<0.001) (**Figure 2E**). Finally, the cut-off for the change in Shannon Entropy (post/baseline) of -0.11 also distinguished two risk groups in terms of PFS, with < -0.11 indicating the good (low-risk) group and > -0.11 the poor (high-risk) group, and these values also correlated with PFS (P<0.001) (**Figure 2F**).

**Figure 3:**
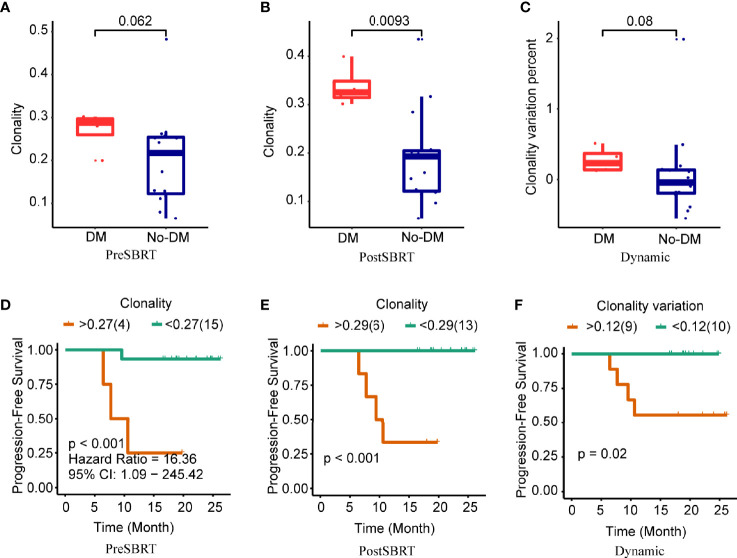
Clonality and treatment outcomes. **(A−C)** Boxplots showing differences in clonality scores in baseline (before-SBRT) samples **(A)**; in samples collected after SBRT **(B)**; and in the difference in clonality between before and after SBRT [clonality after SBRT minus clonality before SBRT)/(clonality before SBRT)], according to whether patients developed metastases after SBRT (DM) or did not (no-DM). **(D−F)** Kaplan–Meier progression-free survival estimates according to clonality cut-off levels for samples obtained before SBRT **(D)**, after SBRT **(E)**, or the percent change in clonality from before to after SBRT **(F)**. Patients with high baseline clonality (i.e., >0.27) had significantly longer progression-free survival; patients with high post-SBRT clonality (>0.29) had longer progression-free survival; and patients with higher percent change in clonality (>0.12) had longer progression-free survival. P values are derived from unadjusted log-rank tests.

Next, clonality was used to estimate TCR oligoclonal levels and evaluated for their associations with the development of metastases. Baseline clonality values were not associated with the development of metastases (P=0.062) (**Figure 3A**). However, clonality values after SBRT were lower for patients who developed metastases after SBRT than for those who did not (P=0.009) (**Figure 3B**); the change in clonality (post/baseline) values seemed lower for those who developed metastases than for those who did not, but this apparent difference was not statistically significant (P=0.08) (**Figure 3C**). For clonality analysis, the use of the same cut-off values also distinguished low-risk versus high-risk patients in terms of PFS (**Figures 3D–F** and **Supplementary Figures 3D–F**). However, the baseline, after-SBRT, and change in the number of unique clones (post/baseline) values did not differ for those who did or did not develop metastases (**Supplementary Figure 4**). Collectively, these findings suggest that the combination of paired peripheral blood CDR3 diversity changes after SBRT provides valuable prognostic information.

### Differences in V and J Gene Usage in T Cells Among Patients With or Without Metastasis After SBRT

**Figure 4:**
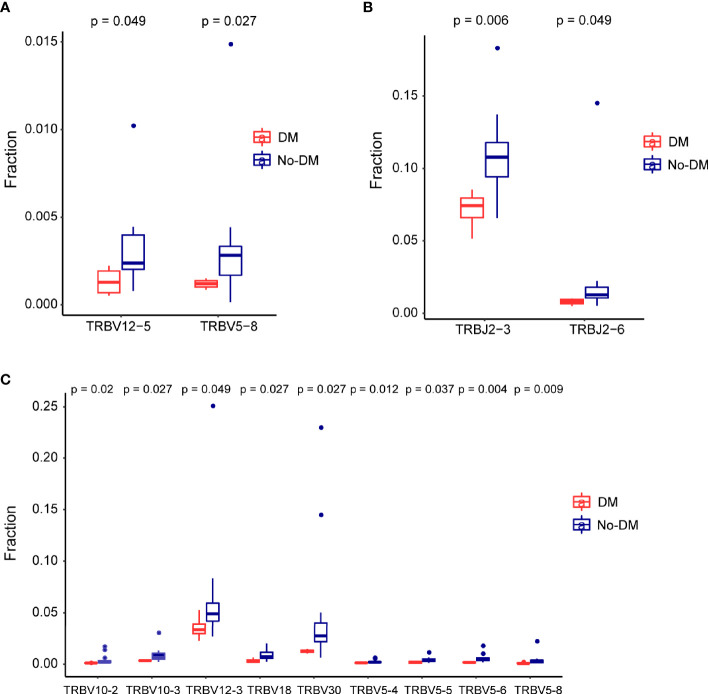
Differences in the T-cell receptor β gene segments in patients with or without failure after SBRT. **(A)** Two segments from the V gene (TRBV12−5 and TRBV5−8) were expressed differently in baseline (before-SBRT) peripheral blood samples according to whether patients did (blue, DM) or did not develop metastases after SBRT (red, non-meta). **(B, C)** Two J segments **(B)** and 9 V segments **(C)** were expressed differently in post-SBRT samples according to whether patients did (blue, metastases) or did not develop metastases after SBRT (red, No-DM).

**Figure 5:**
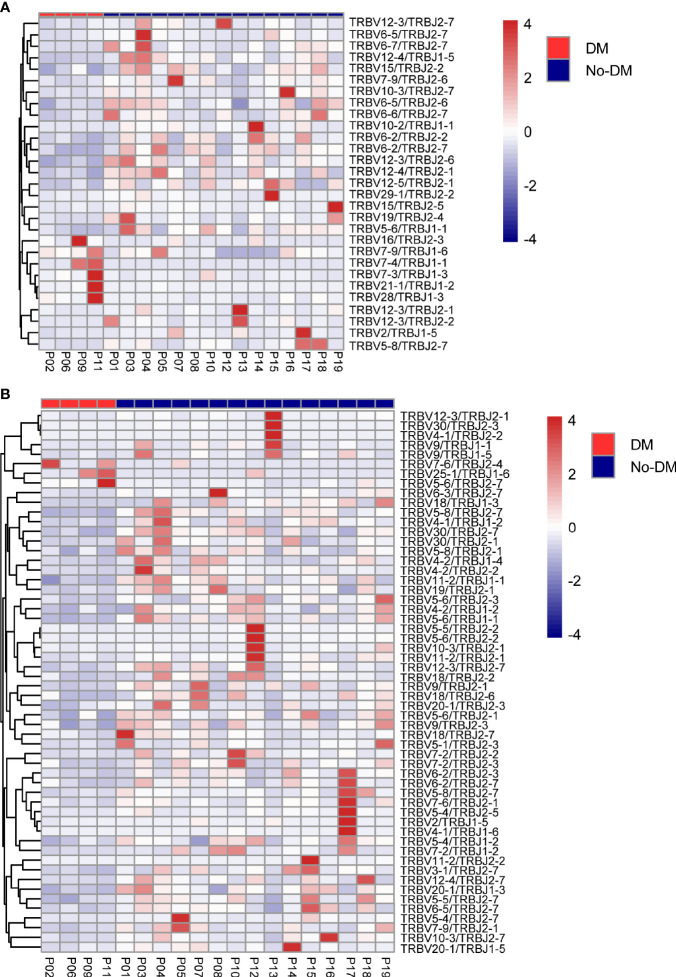
Heatmaps of changes in V and J gene frequencies. **(A)** Heatmaps show significant differences in V-J paring patterns in samples obtained before SBRT according to whether patients did (red) or did not (blue) develop metastases after SBRT. **(B)** Heatmaps show significant differences in V-J paring patterns in samples obtained after SBRT according to whether patients did (red) or did not (blue) develop metastases after SBRT. Color-bar intensity reflects mean proportions of TCR repertoire in the with- or without-metastases groups.

After establishing the sequence of VDJ genes, we counted the expression of recombinant genes in each read, which can represent the relative number of each T cell receptor variable (TRBV). Both the Vβ and Jβ fragments were found to show preferential usage in patients who did or did not develop metastases: in the baseline (before-SBRT) samples, 2 Vβ fragments (TRBV12-5, TRBV5-8) (**Figure 4A**) and 0 Jβ fragments showed different usage frequencies for patients who did or did not develop metastases; in the after-SBRT samples, 2 Jβ fragments (TRBJ2-3, TRBJ2-6) (**Figure 4B**) and 9 Vβ fragments (TRBV10-2, TRBV10-3, TRBV12-3, TRBV18, TRBV30, TRBV5-4, TRBV5-5, TRBV5-6, TRBV5-8) (**Figure 4C**) showed differences in the with-metastases versus no-metastases groups. Furthermore, in the baseline sample, expression of 29 paired V-J genes was different in the with-metastases and no-metastases groups (**Figure 5A**), and in the after-SBRT samples, 56 V-J genes were expressed differently according to metastasis or no metastasis (**Figure 5B**). These findings suggest that the TCR repertoire in peripheral blood samples from patients who develop metastases after SBRT for stage I NSCLC differs in several respects from the TCR repertoire of patients who do not develop metastases.

## Discussion

Radiotherapy is known to affect the immune system, and its ability to recruit antigen-specific T cells has become a topic of significant interest. Preclinical studies have established that abscopal effects are driven by antigen-specific CD8+ T effector cells (31). However, characterizing radiation-induced immunologic effects in patients with cancer has been challenging. Here we report that SBRT induced variations in the diversity of the TCR repertoire in patients with stage I NSCLC, and we further found that TCR diversity could predict clinical outcomes after SBRT. The TCR repertoire seems to have evolved from before to after SBRT, and changes in TCR diversity were also found to be associated with clinical outcomes, suggesting that aspects of the TCR repertoire in PBMCs could be useful as a predictive biomarker for SBRT.

The spectrum of TCR epitopes responsible for tumor neoantigen recognition is diverse owing to the random formation of neoantigens, which are derived from millions of individualized genetic alterations (32). Thus even tumors with similar histologic origins can carry diverse “genomic landscapes” (33). TCR diversity presumably would reflect the probability of neoantigen recognition. The host immune system needs to maintain a diversified TCR repertoire to recognize the variety of tumor neoantigens (34). Preclinical findings suggest that radiotherapy leads to T-cell infiltration at the irradiated site (35); however, the TCR landscape remains dominated by the polyclonal expansion of preexisting T-cell clones. Radiotherapy enhances T-cell trafficking to locally treated tumor sites and augments preexisting anticancer T-cell responses with the capacity to mediate regression of out-of-field tumor lesions when delivered in combination with anti-PD1 therapy (20). A particularly intriguing finding was the discovery of changes in the peripheral T cell repertoire of a patient with metastatic NSCLC who received radiation therapy to a metastatic focus in combination with ipilimumab, and subsequently achieved a complete response to therapy. Mechanistically, this combination treatment with non-ablative radiation therapy and CTLA4 blockade led to increased density of tumor-infiltrating lymphocytes, increased CD8/CD4 ratio, and broadening of the TCR repertoire (36). Also notable were the enrichment of T-cell clones with increased frequency of treatment-specific “public” TCRs and diversification of CDR3 of TCR-β in the T-cell infiltrates from treated tumors. Collectively, these preclinical findings suggest that in the setting of CTLA4 blockade, radiation therapy broadens the TCR repertoire, an essential step to generating effective immune responses for tumor rejection. We further found in the current study that after SBRT, the number of unique TCR clones was lower after SBRT than before. However, no differences were found in the Shannon Entropy from before to after SBRT, implying that some fraction of TCR clones might experience heterogeneous expansion and others might be eliminated under therapeutic pressure.

We also examined correlations between the TCR repertoire, in particular CDR3 diversity, and prognosis in patients with early-stage NSCLC treated with SBRT. Although SBRT is an established treatment option for managing stage I NSCLC, out-of-field failure is the most common mode of failure (both regional and distant). In our study, 4 patients (21%) developed distant metastasis after SBRT, and the remaining 15 patients had no disease progression. The lower Shannon Entropy in the developed metastases group seems to have indicated a lower TCR diversity and a weak immune system. The difference in TCR diversity (post/baseline) was also associated with metastasis, whereas a noticeable increase in diversity (post/baseline) suggested disease control. These findings suggest that the combination of peripheral-blood CDR3 diversity changes after SBRT provides valuable prognostic information for the early-stage of NSCLC patients.

Our results demonstrate a clear association between repertoire diversity and distant metastasis among patients receiving SBRT. The effects of immunotherapy on the peripheral TCR repertoire and overall survival have been described previously for melanoma, pancreatic cancer, and urothelial cancer (37–40). Nevertheless, conclusions regarding the beneficial aspects of the TCR repertoire are inconsistent and sometimes contradictory, and the association of TCR repertoire metrics with clinical outcomes in these studies has yielded mixed results (41). Together, these inconsistencies highlight the need for more precise studies of specific tumor types. To our knowledge, this is the first study to examine the characteristics of the peripheral-blood TCR repertoire and clinical outcomes in patients with early-stage NSCLC treated with SBRT. Our results and those of others suggest that monitoring changes in functional T cells or their TCR repertoire may be a useful complementary and potential surrogate biomarker for the early identification of patients benefiting from SBRT when a pre-SBRT predictor is not available.

In this study, we used powerful immune repertoire sequencing technology to exhaustively analyze the TCR CDR3β repertoires of patients with early-stage NSCLC before and after SBRT. Interestingly, patients who did or did not develop metastases showed different TCR repertoires in terms of V/J segment usage and sequence, chiefly a significant difference in frequencies of the V gene (TRBV12-5, TRBV5-8) in samples taken before SBRT and differences in the frequencies of both the V gene (TRBV10-2, TRBV10-3, TRBV12-3, TRBV18, TRBV30, TRBV5-4, TRBV5-5, TRBV5-6, TRBV5-8) and the J gene (TRBJ2-3, TRBJ2-6) in samples taken after SBRT. There were only 2 differentially V genes between metastatic and non-metastatic groups before SBRT and the overall indexes (Shannon Entropy and clonality) were unremarkable. After SBRT, there were more different V and J genes, and overall indexes were significant differences between metastatic and non-metastatic groups. The consistency of those results indicated that the use of V and J genes may be the internal factors that cause the change of overall index and metastasis. McGranahan et al. explored the influence of heterogeneity in intratumoral neoantigens on antitumor immunity in early-stage NSCLC and determined that tumors with both a high clonal neoantigen burden and low neoantigen intratumor heterogeneity were associated with significantly longer PFS times (42). Our findings imply that the percentage frequencies of V and J gene usage are low in patients who develop metastases relative to those who do not, results that are consistent with our analyses of the Shannon Entropy and clonality and suggest that patients who develop metastases after SBRT have lower TCR diversity and a weaker immune system. Finally, we also analyzed VJ combinations in patients with early-stage NSCLC and found some fixed VJ combinations in both patients with metastases and those without metastases. Whether these specific VJ combinations and the shared clones are related to tumor antigens requires further study.

This study had several limitations, chief among them the small number of patients and short follow-up period. Our data need to be verified in a more extensive and independent cohort with similar inclusion criteria. Additional studies are also needed to assess the TCR specificity of clonally expanded T cells in patients with good prognosis to determine which epitopes are being recognized. Another limitation is that we could not determine if the expanded clones identified in patients with good prognosis were tumor-specific. Nevertheless, our analysis of the essential characteristics and clinical significance of the TCR repertoire in early-stage NSCLC treated with SBRT lead us to propose that high baseline TCR diversity may predict a favorable outcome after SBRT; further monitoring of the TCR repertoire is needed for more accurate predictions. We are currently conducting such studies.

In conclusion, we observed significant changes in the diversity of the TCR repertoire after SBRT, and that the TCR profile and expression of TCR-β V and J genes differed significantly between patients who developed metastases and those who did not. Our results suggest that the effects of SBRT on TCR diversity and TCR profiles may be useful for identifying which patients may be more likely to experience treatment failure after SBRT for early-stage NSCLC.

## Data Availability Statement

The datasets presented in this study can be found in online repositories. The names of the repository/repositories and accession number(s) can be found below: NCBI SRA BioProject, accession no: PRJNA767497.

## Author Contributions

XH, JF, and RY conceived the study and designed the experiments. NP-R and ZL developed the methodology. LW, JZ, and XP acquired the data. PaL, ZZ, and TX did the bioinformatic analysis and performed initial exploratory analysis. XZ and MJ provided insight in methodological approaches and analysis. LW, NP-R and ZL drafted the paper. JZ supervised the study. JW and PeL gave the comments. All authors contributed to the article and approved the submitted version.

## Funding

This study was funded by China Postdoctoral Science Foundation (2018M640465), Jiangsu Provincial High Level Health Talents Project (No. LGY2019076), the Project of Jiangsu Provincial Medical Talent (ZDRCA2016033), National Natural Science Foundation of China (81871873) and National Natural Science Foundation of China (82003232).

## Conflict of Interest

PaL and ZZ were employed by Geneplus-Beijing Institute.

The remaining authors declare that the research was conducted in the absence of any commercial or financial relationships that could be construed as a potential conflict of interest.

## Publisher’s Note

All claims expressed in this article are solely those of the authors and do not necessarily represent those of their affiliated organizations, or those of the publisher, the editors and the reviewers. Any product that may be evaluated in this article, or claim that may be made by its manufacturer, is not guaranteed or endorsed by the publisher.

## Supplementary Material

Supplementary Table 1 Primers for TCR-β sequencing.

Supplementary Figure 1 TCR β sequencing of peripheral blood lymphocytes before and after stereotactic body radiotherapy (SBRT). Genomic DNA from peripheral blood mononuclear cell samples from 19 patients was quantitatively TCR-β-sequenced to compare T-cell clonality from before to after SBRT. Clones increasing in frequency are in blue, those decreasing in frequency are in red, and those with no significant change are in green.

Supplementary Figure 2 The new and loss TCR frequency during SBRT.

Supplementary Figure 3 Receiver operating characteristic curves for the Shannon index. The ROC curves in panels **(A–C)** show the optimal cut points for the Shannon index in samples obtained at baseline (before SBRT) after SBRT, and the change between before and after SBRT (Dynamic). Dots indicate optimal cut-point values as well as sensitivity and specificity.

Supplementary Figure 4 Treatment outcomes versus numbers of unique clones. Boxplots in panels **(A–C)** show differences in clone counts in samples collected before stereotactic body radiation therapy (PreSBRT), samples collected after SBRT (PostSBRT), and the change between before and after SBRT (Dynamic) according to whether patients developed or did not develop distant metastases (DM) after SBRT. Kaplan–Meier survival estimates in panels **(D–F)** show progression-free survival curves for patients with clone counts above and below the identified cutpoints derived from baseline samples (PreSBRT), samples obtained after SBRT (PostSBRT), and the change between before and after SBRT (Dynamic). P values are derived from unadjusted log-rank analyses.
