# Supplementary material for: T-Cell Receptor Profiling and Prognosis After Stereotactic Body Radiation Therapy For Stage I Non-Small-Cell Lung Cancer
Source: Front Immunol. 2021 Oct 18;12:719285. doi: 10.3389/fimmu.2021.719285 (PMC8559517; doi:10.3389/fimmu.2021.719285)
Supplement: Supplementary Table 1 — Primers for TCR-β sequencing. [file Table_1.docx]

**Supplementary Table 1. Primers for TCR-β sequencing.**

| ID | 5’-3’ |
| --- | --- |
| TRBV2F | CAGACGTGTGCTCTTCCGATCTAGATTTCACTCTGAAGATCCGGTCCAC |
| TRBV3-1-F4 | CAGACGTGTGCTCTTCCGATCTAGAAACAGTTCCAAATCGMTTCTCAC |
| TRBV4-1/2/3-F4 | CAGACGTGTGCTCTTCCGATCTAGCAAGTCGCTTCTCACCTGAATG |
| TRBV5-1-F4 | CAGACGTGTGCTCTTCCGATCTAGGCCAGTTCTCTAACTCTCGCTCT |
| TRBV5-4/5/6/8-F4 | CAGACGTGTGCTCTTCCGATCTAGTCAGGTCGCCAGTTCCCTAAYTAT |
| TRBV6-4.1-F | CAGACGTGTGCTCTTCCGATCTAGCACGTTGGCGTCTGCTGTACCCT |
| TRBV6-8/5/1.2-F | CAGACGTGTGCTCTTCCGATCTAGCAGGCTGGTGTCGGCTGCTCCCT |
| TRBV6-9/7/1.1/6-F | CAGACGTGTGCTCTTCCGATCTAGCAGGCTGGAGTCAGCTGCTCCCT |
| TRBV6-4.2-F | CAGACGTGTGCTCTTCCGATCTAGAGTCGCTTGCTGTACCCTCTCAG |
| TRRBV6-2/3-F | CAGACGTGTGCTCTTCCGATCTAGGGGGTTGGAGTCGGCTGCTCCCT |
| TRBV7-2/4/6/7/8-F4 | CAGACGTGTGCTCTTCCGATCTAGGGGATCCGTCTCCACTCTGAMGAT |
| TRBV7-3-F4 | CAGACGTGTGCTCTTCCGATCTAGGGGATCCGTCTCTACTCTGAAGAT |
| TRBV7-9-F4 | CAGACGTGTGCTCTTCCGATCTAGGGGATCTTTCTCCACCTTGGAGAT |
| TRBV9F | CAGACGTGTGCTCTTCCGATCTAGCCTGACTTGCACTCTGAACTAAACCT |
| TRBV10-1-F4 | CAGACGTGTGCTCTTCCGATCTAGCCTCACTCTGGAGTCTGCTGCC |
| TRBV10-2/3-F4 | CAGACGTGTGCTCTTCCGATCTAGCCTCACTCTGGAGTCMGCTACC |
| TRBV11-1/2/3-F4 | CAGACGTGTGCTCTTCCGATCTAGGCAGAGAGGCTCAAAGGAGTAGACT |
| TRBV12-3.2/5.2 | CAGACGTGTGCTCTTCCGATCTAGGAAGGTGCAGCCTGCAGAACCCAG |
| TRBV12-3.1/4/5.1-F | CAGACGTGTGCTCTTCCGATCTAGGAAGATCCAGCCCTCAGAACCCAG |
| TRBV13-F4 | CAGACGTGTGCTCTTCCGATCTAGTCGATTCTCAGCTCAACAGTTC |
| TRBV14F | CAGACGTGTGCTCTTCCGATCTAGGGAGGGACGTATTCTACTCTGAAGG |
| TRBV15F | CAGACGTGTGCTCTTCCGATCTAGTTCTTGACATCCGCTCACCAGG |
| TRBV16-F2 | CAGACGTGTGCTCTTCCGATCTAGCTGTAGCCTTGAGATCCAGGCTACGA |
| TRBV18-F4 | CAGACGTGTGCTCTTCCGATCTAGTAGATGAGTCAGGAATGCCAAAG |
| TRBV19F | CAGACGTGTGCTCTTCCGATCTAGTCCTTTCCTCTCACTGTGACATCGG |
| TRBV20-1-F4 | CAGACGTGTGCTCTTCCGATCTAGAACCATGCAAGCCTGACCTT |
| TRBV24-1-F2 | CAGACGTGTGCTCTTCCGATCTAGCTCCCTGTCCCTAGAGTCTGCCAT |
| TRBV25-1F | CAGACGTGTGCTCTTCCGATCTAGGCCCTCACATACCTCTCAGTACCTC |
| TRBV27-1 | CAGACGTGTGCTCTTCCGATCTAGGATCCTGGAGTCGCCCAGC |
| TRBV28 | CAGACGTGTGCTCTTCCGATCTAGATTCTGGAGTCCGCCAGC |
| TRBV29-1-F4 | CAGACGTGTGCTCTTCCGATCTAGAACTCTGACTGTGAGCAACATGAG |
| TRBV30-F5 | CAGACGTGTGCTCTTCCGATCTAGCAGATCAGCTCTGAGGTGCCCCA |
| TRBJ1.1-R2 | CTACACGACGCTCTTCCGATCTCTTACCTACAACTGTGAGTCTGGTG |
| TRBJ1.2R | CTACACGACGCTCTTCCGATCTCTTACCTACAACGGTTAACCTGGTC |
| TRBJ1.3R | CTACACGACGCTCTTCCGATCTCTTACCTACAACAGTGAGCCAACTT |
| TRBJ1-4 | CTACACGACGCTCTTCCGATCTAAGACAGAGAGCTGGGTTCCACT |
| TRBJ1.5R | CTACACGACGCTCTTCCGATCTCTTACCTAGGATGGAGAGTCGAGTC |
| TRBJ1.6R | CTACACGACGCTCTTCCGATCTCATACCTGTCACAGTGAGCCTG |
| TRBJ2.1R | CTACACGACGCTCTTCCGATCTCCTTCTTACCTAGCACGGTGA |
| TRBJ2.2R | CTACACGACGCTCTTCCGATCTCTTACCCAGTACGGTCAGCCT |
| TRBJ2.3R | CTACACGACGCTCTTCCGATCTCCGCTTACCGAGCACTGTCAG |
| TRBJ2-4 | CTACACGACGCTCTTCCGATCTAGCACTGAGAGCCGGGTCC |
| TRBJ2.5-R2 | CTACACGACGCTCTTCCGATCTCGAGCACCAGGAGCCGCGT |
| TRBJ2.6R | CTACACGACGCTCTTCCGATCTCTCGCCCAGCACGGTCAGCCT |
| TRBJ2.7-R2 | CTACACGACGCTCTTCCGATCTCTTACCTGTGACCGTGAGCCTG |
