# Supplementary figures and images for: T-Cell Receptor Profiling and Prognosis After Stereotactic Body Radiation Therapy For Stage I Non-Small-Cell Lung Cancer
Source: Front Immunol. 2021 Oct 18;12:719285. doi: 10.3389/fimmu.2021.719285 (PMC8559517; doi:10.3389/fimmu.2021.719285)

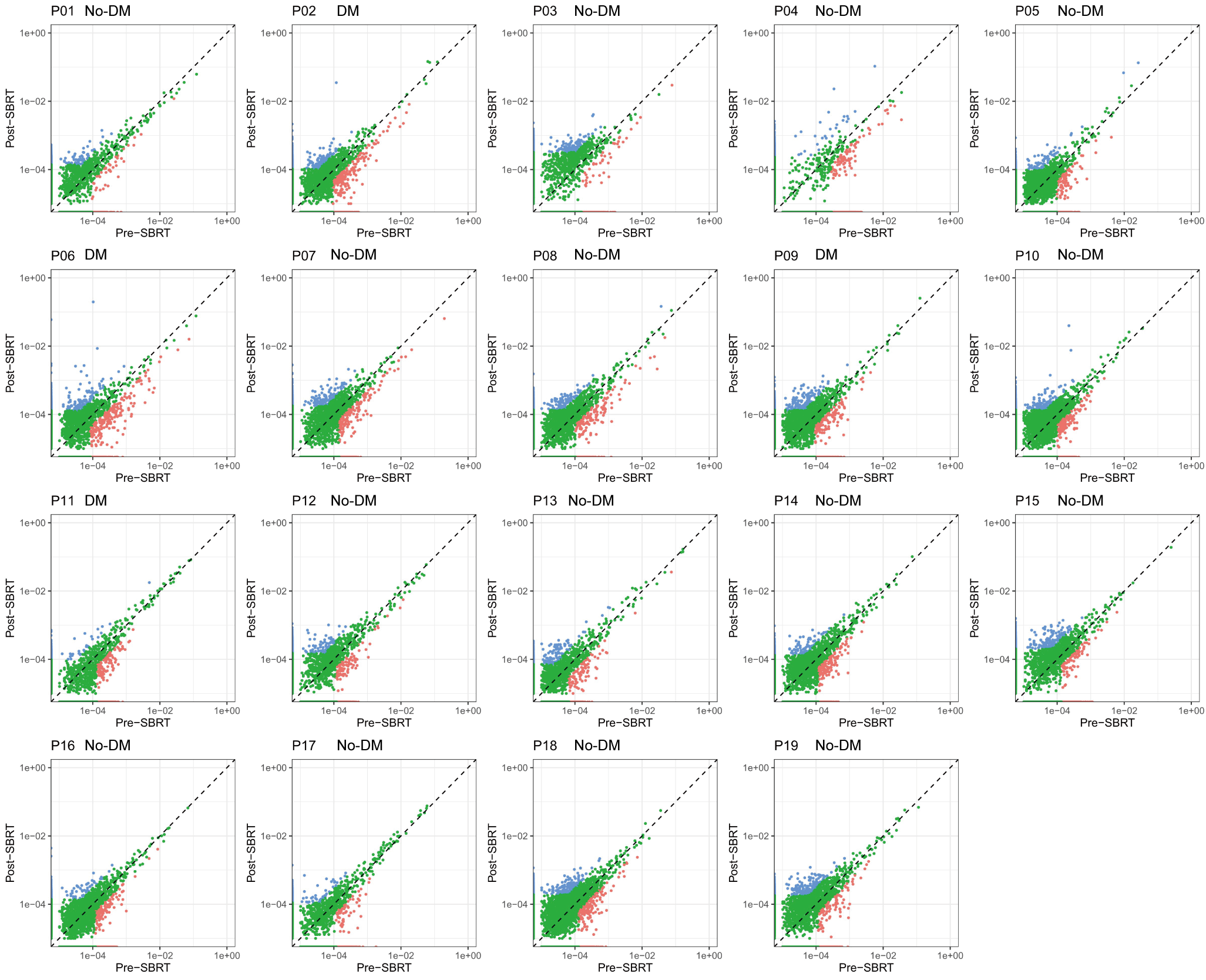

Supplement: Supplementary Figure 1 — TCR β sequencing of peripheral blood lymphocytes before and after stereotactic body radiotherapy (SBRT). Genomic DNA from peripheral blood mononuclear cell samples from 19 patients was quantitatively TCR-β-sequenced to compare T-cell clonality from before to after SBRT. Clones increasing in frequency are in blue, those decreasing in frequency are in red, and those with no significant change are in green. [file Image_1.tif]

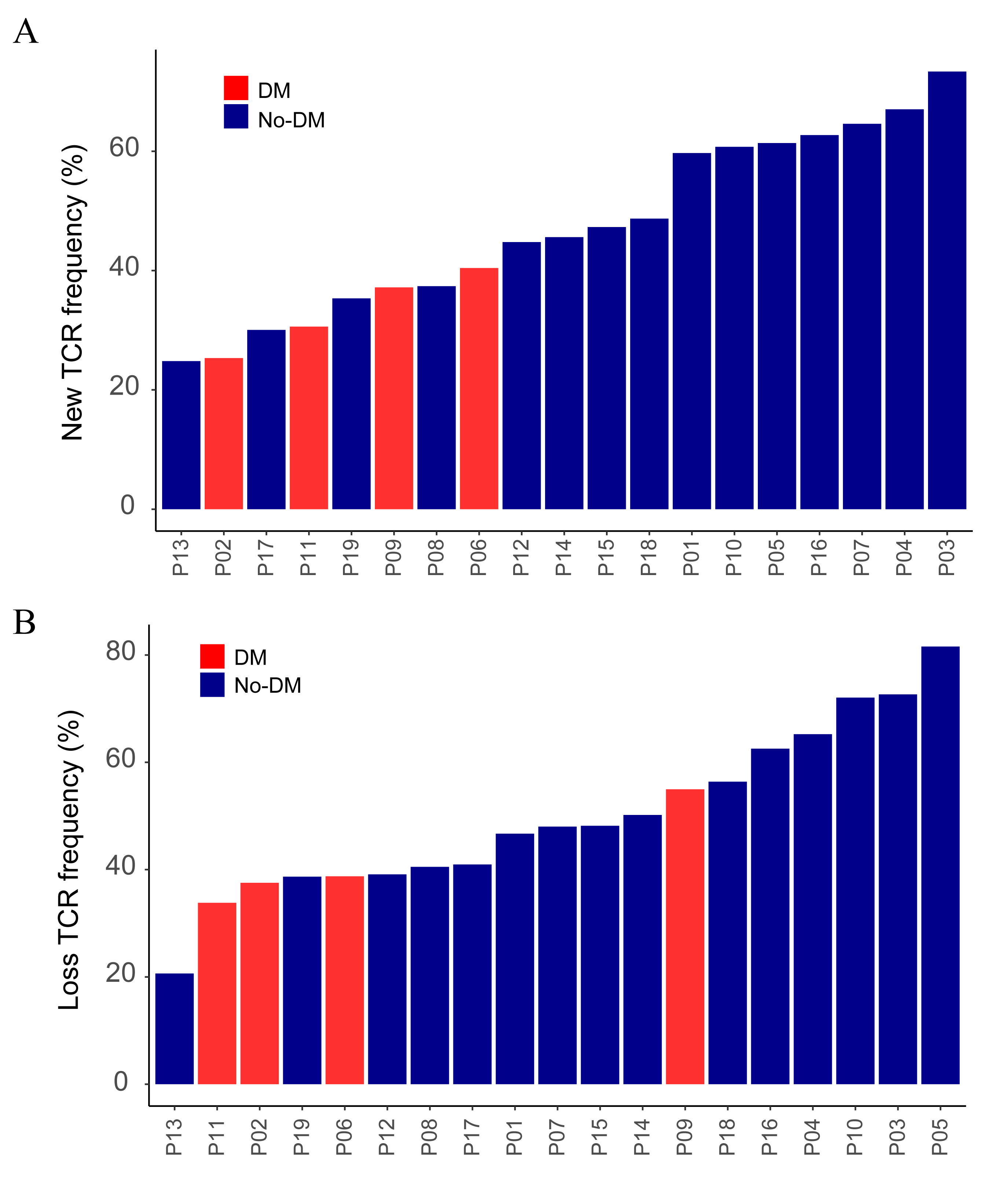

Supplement: Supplementary Figure 2 — The new and loss TCR frequency during SBRT. [file Image_2.tif]

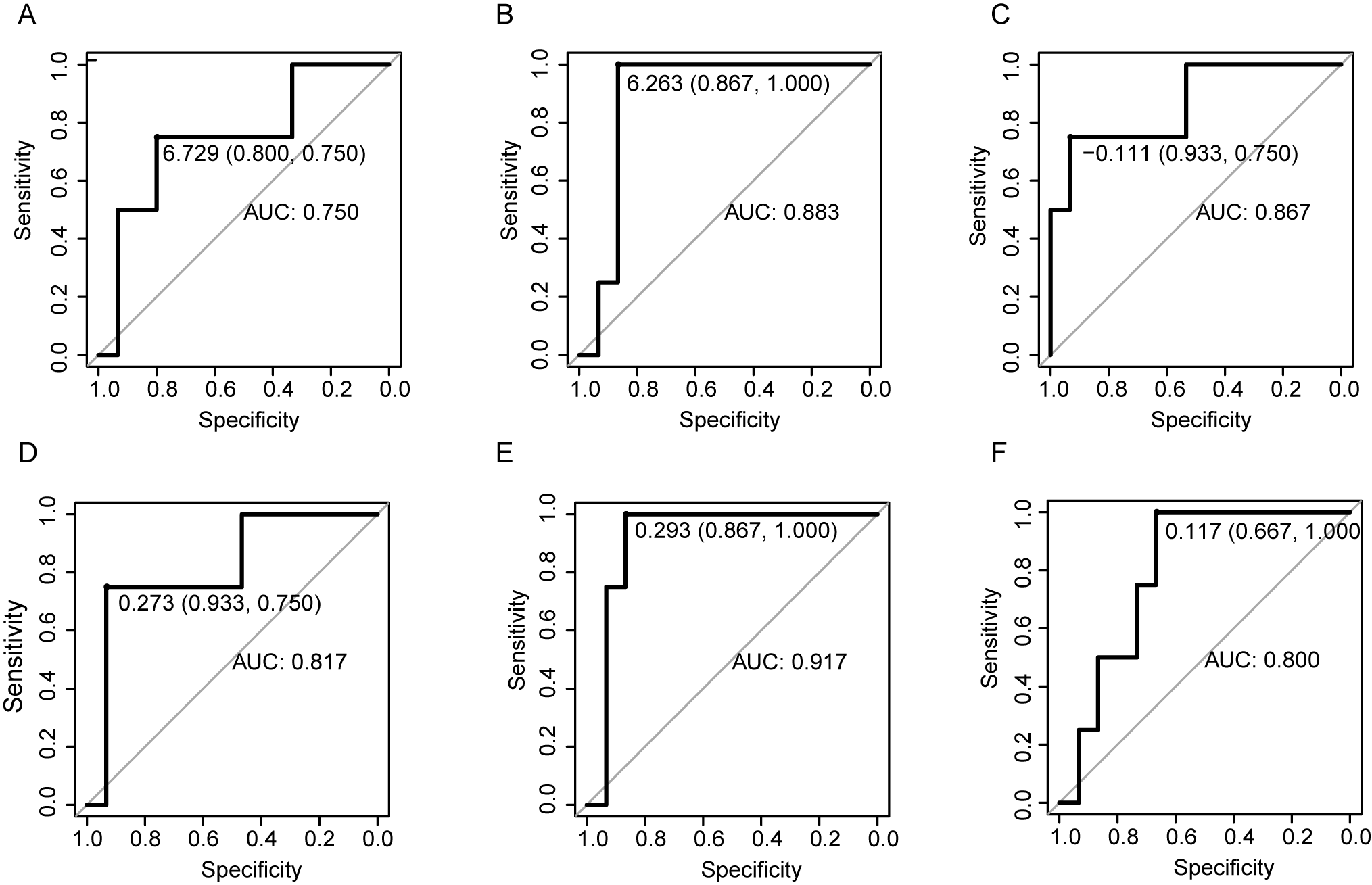

Supplement: Supplementary Figure 3 — Receiver operating characteristic curves for the Shannon index. The ROC curves in panels (A–C) show the optimal cut points for the Shannon index in samples obtained at baseline (before SBRT) after SBRT, and the change between before and after SBRT (Dynamic). Dots indicate optimal cut-point values as well as sensitivity and specificity. [file Image_3.tif]

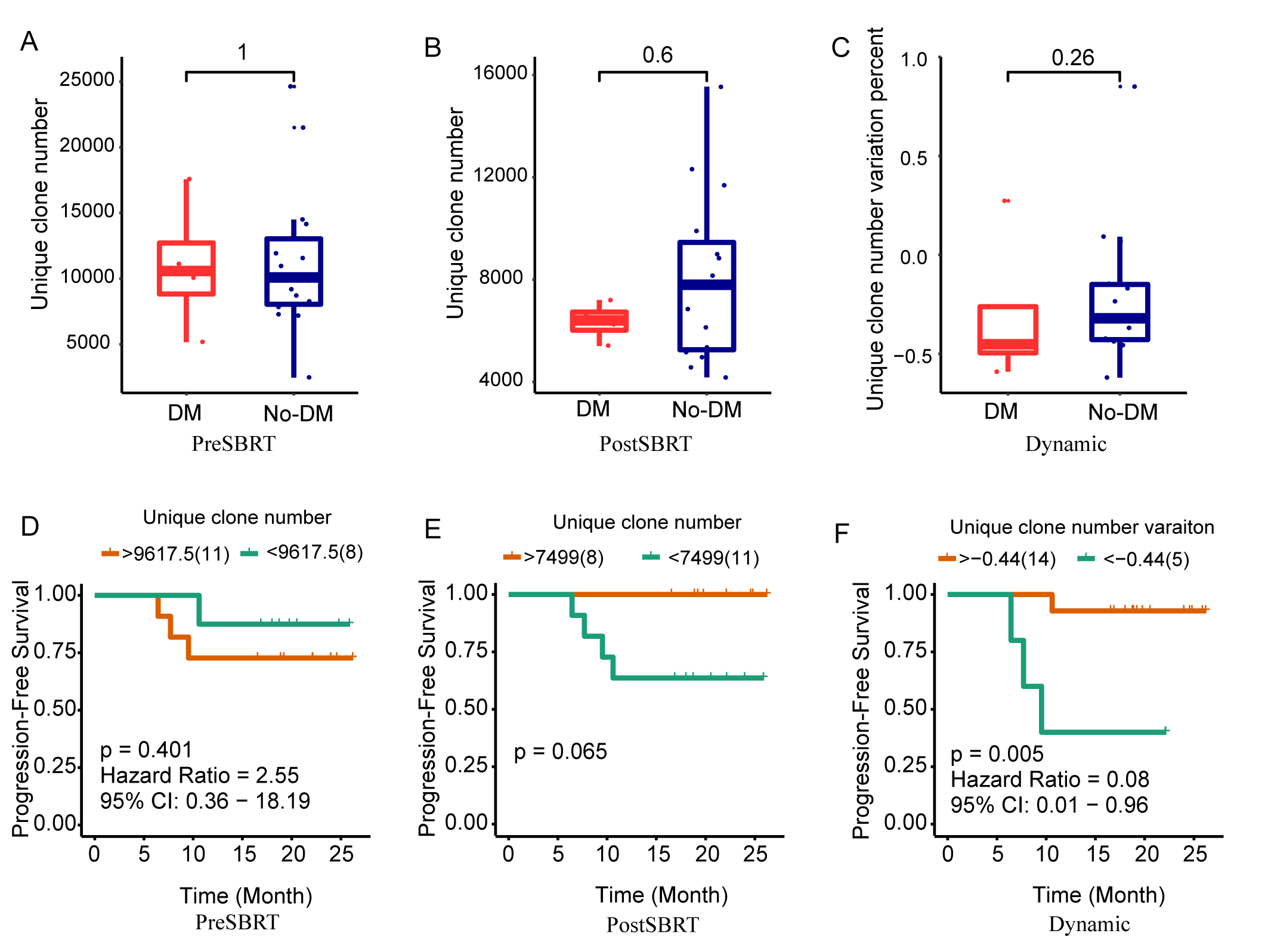

Supplement: Supplementary Figure 4 — Treatment outcomes versus numbers of unique clones. Boxplots in panels (A–C) show differences in clone counts in samples collected before stereotactic body radiation therapy (PreSBRT), samples collected after SBRT (PostSBRT), and the change between before and after SBRT (Dynamic) according to whether patients developed or did not develop distant metastases (DM) after SBRT. Kaplan–Meier survival estimates in panels (D–F) show progression-free survival curves for patients with clone counts above and below the identified cutpoints derived from baseline samples (PreSBRT), samples obtained after SBRT (PostSBRT), and the change between before and after SBRT (Dynamic). P values are derived from unadjusted log-rank analyses. [file Image_4.tif]
